# Who Will Rise up and Answer?

**Published:** 1899-07

**Authors:** 


### WHO WILL RISE UP AND ANSWER?

WHY don't that Allegheny County Veterinary Association rear its head high enough to be seen?

Why don't New York State veterinarians demand a revision of their laws regulating veterinary practice, that will give some ray of hope of a National Board of Veterinary Medical Examiners?

Why don't some one in Michigan stand up and defend the new veterinary law in that State from the open attacks made upon it by members of the profession in that Commonwealth?

When does Maryland look for reciprocity, with its own law so constructed as to require examination of those with licenses from other States, and the law disapproved of by a large number of her most prominent graduates?

Will Massachusetts ever regain the strong place she once held so proudly in the field of sanitary progress along veterinary lines?

---

THE concessions made by the last Congress are only the entering wedge to better things, and the coming session of our national legislature must find us more thoroughly grounded in our determination that this most progressive land in the world shall no longer have the meanest and most-to-be-despised army veterinary service.

---

### MARRIAGES.

At Starkville, Miss., July 10, 1899, Dr. George E. Nesom to Miss Bessie O'Brien. Dr. Nesom is a graduate of the Veterinary Department of the Iowa Agricultural College, Class of 1898, and now located at Clemson College, South Carolina, as Professor of Veterinary Medicine and Veterinarian to the Experiment Station. The newly-married couple will be at home at Clemson College after August 2, 1899.

Dr. Samuel G. Hendren, of the Bureau of Animal Industry, stationed at Indianapolis, Ind , was recently united in marriage to Miss Marion Weber, of Lewistown, Pa.

Dr. John P. O'Leary, of the Bureau of Animal Industry at Boston, Mass., was recently united in marriage to Miss Lillian Milsom, of Buffalo, N. Y.

